# A LINE-1 Insertion in DLX6 Is Responsible for Cleft Palate and Mandibular Abnormalities in a Canine Model of Pierre Robin Sequence

**DOI:** 10.1371/journal.pgen.1004257

**Published:** 2014-04-03

**Authors:** Zena T. Wolf, Elizabeth J. Leslie, Boaz Arzi, Kartika Jayashankar, Nili Karmi, Zhonglin Jia, Douglas J. Rowland, Amy Young, Noa Safra, Saundra Sliskovic, Jeffrey C. Murray, Claire M. Wade, Danika L. Bannasch

**Affiliations:** 1 Department of Population Health and Reproduction, School of Veterinary Medicine, University of California, Davis, Davis, California, United States of America; 2 Department of Pediatrics, University of Iowa, Iowa City, Iowa, United States of America; 3 Department of Surgical and Radiological Sciences, School of Veterinary Medicine, University of California, Davis, Davis, California, United States of America; 4 State Key Laboratory of Oral Diseases, West China Hospital of Stomatology, Sichuan University, Chengdu, China; 5 Department of Cleft Lip and Palate Surgery, West China Hospital of Stomatology, Sichuan University, Chengdu, China; 6 Center for Molecular and Genomic Imaging, University of California, Davis, Davis, California, United States of America; 7 Department of Animal Science, University of California, Davis, Davis, California, United States of America; 8 Faculty of Veterinary Science, University of Sydney, Sydney, New South Wales, Australia; Stanford University School of Medicine, United States of America

## Abstract

Cleft palate (CP) is one of the most commonly occurring craniofacial birth defects in humans. In order to study cleft palate in a naturally occurring model system, we utilized the Nova Scotia Duck Tolling Retriever (NSDTR) dog breed. Micro-computed tomography analysis of CP NSDTR craniofacial structures revealed that these dogs exhibit defects similar to those observed in a recognizable subgroup of humans with CP: Pierre Robin Sequence (PRS). We refer to this phenotype in NSDTRs as CP1. Individuals with PRS have a triad of birth defects: shortened mandible, posteriorly placed tongue, and cleft palate. A genome-wide association study in 14 CP NSDTRs and 72 unaffected NSDTRs identified a significantly associated region on canine chromosome 14 (24.2 Mb–29.3 Mb; p_raw_ = 4.64×10^−15^). Sequencing of two regional candidate homeobox genes in NSDTRs, distal-less homeobox 5 (DLX5) and distal-less homeobox 6 (DLX6), identified a 2.1 kb LINE-1 insertion within DLX6 in CP1 NSDTRs. The LINE-1 insertion is predicted to insert a premature stop codon within the homeodomain of DLX6. This prompted the sequencing of DLX5 and DLX6 in a human cohort with CP, where a missense mutation within the highly conserved DLX5 homeobox of a patient with PRS was identified. This suggests the involvement of DLX5 in the development of PRS. These results demonstrate the power of the canine animal model as a genetically tractable approach to understanding naturally occurring craniofacial birth defects in humans.

## Author Summary

Cleft palate is one of the most commonly occurring birth defects in children, and yet its cause is not completely understood. In order to better understand cleft palate we have turned to man's best friend, the domestic dog. Common breeding practices have made the dog a unique animal model to help understand the genetic basis of naturally occurring birth defects. A genome-wide association study of Nova Scotia Duck Tolling Retrievers with naturally occurring cleft palate led to the investigation of two homeobox genes, DLX5 and DLX6. Dogs with this mutation also have a shortened lower jaw, which resembles those who have Pierre Robin Sequence (PRS). Investigation into people with PRS identifies a mutation within a highly conserved and functional region of DLX5 that may contribute to the development of PRS. This exemplifies how the dog will help us better understand common birth defects.

## Introduction

Cleft palate (CP) is one of the most commonly occurring craniofacial birth defects, affecting approximately 1 in 1500 live human births in the United States [1]. Children born with CP may develop hearing loss, difficulties with speech and eating, and may be at an increased risk for psychiatric disorders and neurological deficits [2]–[4]. CP occurs when there is a failure in the formation of the secondary palate, which makes up all of the soft palate and majority of the hard palate. Secondary palate development is conserved across mammalian species and proceeds through highly regulated sequential steps: palatal shelf growth, elevation, fusion, and cell differentiation (reviewed in [5]). Disruptions in any of these pathways may cause a cleft palate and lead to the phenotypic spectrum of CP cases that is observed. CP may occur alone (nonsyndromic) or with other abnormalities (syndromic).

Pierre Robin sequence (PRS, OMIM 261800) is a heterogeneous and phenotypically variable subgroup of CP that affects 1 in 8500 live human births [6]. PRS is characterized by a cleft palate, shortened mandible (micrognathia), and posteriorly placed tongue (glossoptosis). PRS is thought to be the result of a sequence of events caused by the primary defect, micrognathia [7]. The etiology of PRS is still largely unknown and highly variable. PRS may occur alone or as part of a syndrome, such as Stickler syndrome, Velocardiofacial syndrome, and Treacher Collins syndrome [8], [9]. A high incidence within families and among twins suggests a genetic etiology. Familial aggregation with an autosomal dominant mode of inheritance has been observed with translocations of 17q24 and a reduction of SOX9 and KCNJ2 expression [10], [11]. PRS also occurs at a high incidence among families with cleft lip and palate [12], [13]. However, monozygotic twins discordant for PRS are also observed, suggesting that PRS may be a result of the twinning process or of mandibular constraint in utero [13], [14].

In an effort to understand the genetic basis of craniofacial birth defects such as PRS, we used a relatively unconventional model organism, the domestic dog. Dogs have naturally occurring birth defects, with inherited orofacial clefts that resemble those observed in humans [15]–[17]. Domestication and subsequent pedigreed breed creation from a small number of founders has led to a unique genetic background, resulting in a small number of genetic variants being responsible for the broad phenotypic diversity observed [18]. Compared to humans, dogs are amendable to association-based mapping studies with a small number of samples due to their relatively long linkage disequilibrium blocks within breeds [19]. Here, we demonstrate how a naturally occurring model of PRS in the Nova Scotia Duck Tolling Retriever (NSDTR) breed, characterized by CP and relative micrognathia, led to the identification of both the first mutation responsible for cleft palate in dogs and candidate genes for PRS in people.

## Results

### Genome-Wide Association

**Figure 1:**
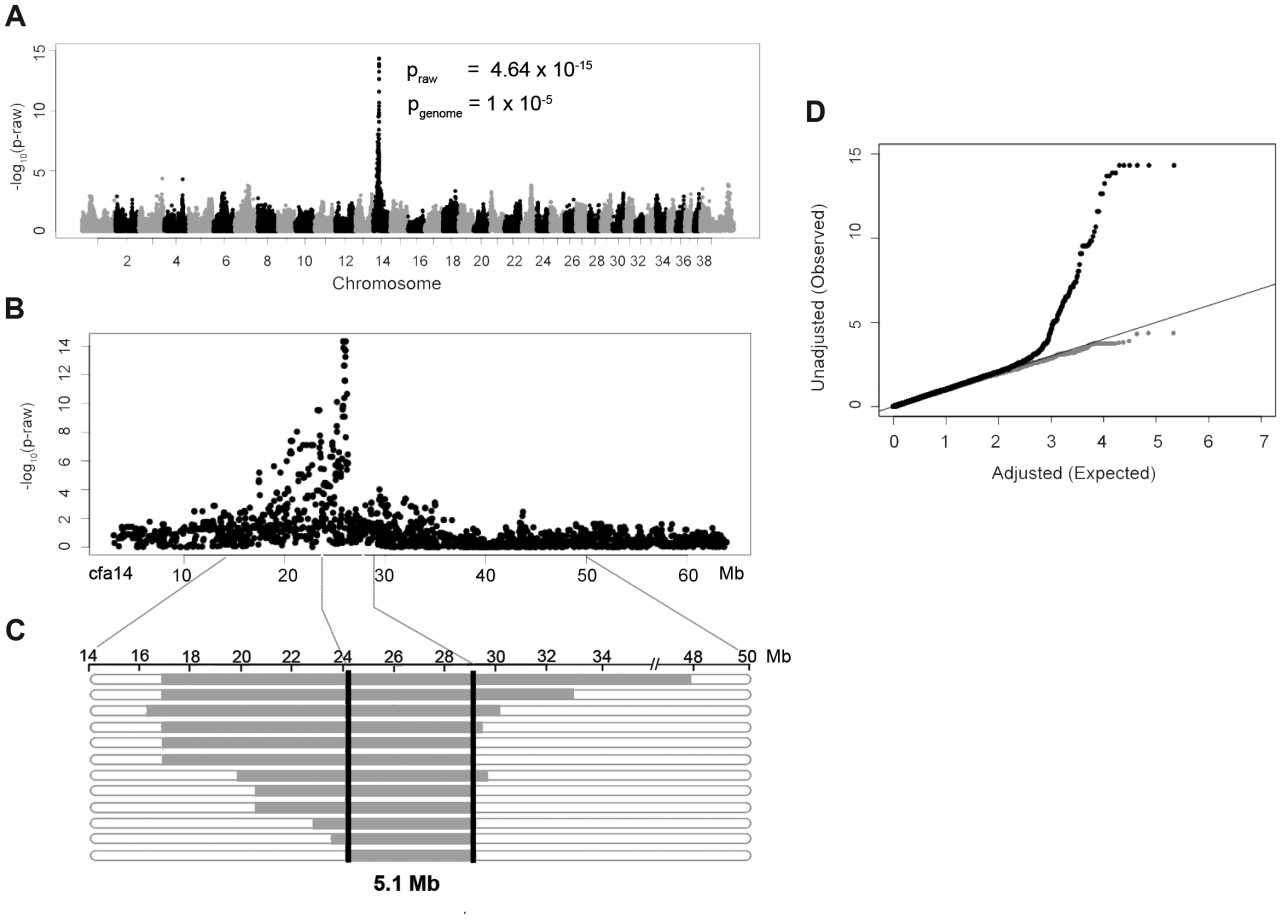
Genome-wide association study results of NSDTRs with CP. A. Manhattan plot of −log_10_ of raw p-values by chromosome. The p_genome_ value is the p-value after 100000 permutations. The lowest p_genome_ is found across 8 SNPs on cfa14: 25822897, 25832747, 25847915, 25854827, 25868609, 25995782, 26023199, and 26082330. B. Plot of the raw p-values by Mb on cfa14 depicting the associated region. C. Observed haplotypes in 12 CP NSDTRs. Horizontal bars represent haplotypes from the 12 CP NSDTRs with associated haplotype, with runs of homozygosity in grey. The critical interval is defined by the shared homozygous haplotype denoted by the black bars (cfa14. 24189817-29319290). The 2 CP NSDTRs without the associated haplotype are not included in this figure. D. Quantile-Quantile plot of genome-wide association results. Black dots represent the observed versus expected p-values of all SNPs (λ = 1.05). Grey dots represent the observed versus expected p-values after removal of all SNPs on cfa14. (λ = 1.02). The solid grey line represents the null hypothesis: observed p-values equal expected p-values.

DNA samples were collected from 14 NSDTRs that had clefts of the hard and soft palate. To identify loci associated with the CP phenotype in the NSDTR, a genome-wide association was performed in 14 CP NSDTR cases and 72 controls across ∼173,000 SNPs. After quality control, 109,506 SNPs remained. Chi-square analysis of the remaining SNPs identified an associated region on canine chromosome 14 (cfa14; Figures 1A and 1B). A homozygous 5.1 Mb haplotype was identified (24.2 Mb to 29.3 Mb) in 12 of the 14 CP NSDTR cases (Figure 1C). This homozygous haplotype is absent in all 72 controls. Parents (n = 5) and littermates (n = 6) of the 12 CP NSDTRs were heterozygous for the associated haplotype, suggesting a recessive mode of inheritance.

Quantile-quantile (Q-Q) plots and a genomic inflation factor of 1.05 indicate that there is no underlying population stratification (Figure 1D). To confirm that only the association is responsible for the deviation from the line of the null hypothesis, SNPs on cfa14 were removed and the Q-Q plot regenerated. There is little evidence of population stratification with a recalculated genomic inflation factor of 1.02.

Using microsatellite markers from the cfa14 interval, linkage analysis was performed on a subset of CP NSDTRs with the associated haplotype and available family members (n = 8). LOD scores were calculated under a fully penetrant recessive mode of inheritance (Table S1). A significant LOD score of 3.18 was obtained with a recombination fraction (Θ) of zero at cfa14.25006375, further confirming the association.

### CP and Mandibular Abnormalities in NSDTRs

**Figure 2:**
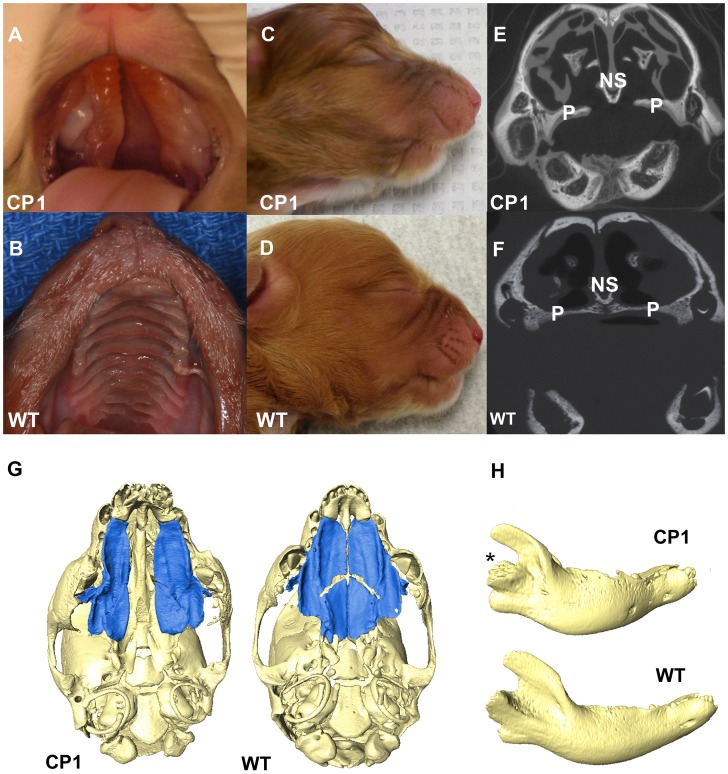
Phenotype of neonatal CP1 NSDTRs. A. Neonatal CP1 NSDTR with an extensive cleft of the hard and soft palate. B. Neonatal NSDTR with a normal palate (WT). C. Lateral view of CP1 head exhibiting relative mandibular brachygnathia. D. Lateral view of WT head with a normal jaw relationship. E. Coronal CT image depicting the failure of the palatine processes and nasal septum to fuse in CP1 NSDTRs. F. Coronal CT image depicting midline fusion of palatal process and nasal septum in WT. P – Palatine process, NS – Nasal septum G. 3D reconstruction of microCT imaging of CP1 and WT skulls with mandibles removed. CP1 skull shows abnormally shaped palatine process and palatine bones. Bones colored blue are the palatine processes and palatine bones. WT skull shows anatomical location of normal palatine sutures and shape of palatine processes and palatine bones. H. 3D reconstruction of mandibles depicting abnormal angulation of the condylar process (*) in CP1 mandibles compared to WT mandibles.

All 14 CP NSDTRs had clefts of the hard and soft palate (Figures 2A and 2B), but the 12 CP NSDTRs with the associated haplotype exhibited a specific phenotype, which we designate CP1 (Cleft Palate 1). Micro-computed tomographic (micro-CT) imaging was performed to investigate additional craniofacial abnormalities on available skulls from 4 neonatal CP1 NSDTRs and 3 neonatal normal NSDTRs with the homozygous wildtype haplotype. One of the mandibles from the 4 CP1 NSDTRs was unavailable for imaging. Uneven alignment of the upper and lower jaw was noted in the 3 CP1 NSDTRs when compared to the 3 normal NSDTRs indicating relative mandibular brachygnathia (Figures 2C and 2D). 3D measurements of the mandible length indicated that the CP1 NSDTRs had relatively shorter mandibles by an average of 5.46 mm when compared to the normal NSDTRs (Table S2). In the 4 cases, clefts were characterized by abnormal or missing palatine fissures, missing or small palatine processes of the maxilla, and small, missing, or abnormally shaped palatine bones (Figures 2E–2G). The nasal septum was absent or poorly developed. In the three CP1 NSDTR with mandibles, variation from the normal angulation of the condylar process was observed (Figure 2H). In two of these cases, an abnormal angle of the mandibular head of the condylar process was observed. One case had an additional general asymmetry of the entire craniofacial complex. Based on the phenotypic findings, we hypothesize that the 12 CP1 NSDTRs are animal models for cleft palate and micrognathia disorders.

Although unavailable for micro-CT imaging, the 2 CP cases without the associated haplotype exhibited a normal jaw relationship with no relative mandibular brachygnathism. Since these dogs are phenotypically different, they were excluded from the rest of the analysis.

### Sanger Sequencing of DLX5 and DLX6

Located within the interval defined by the genome-wide association study are 21 genes (Table S3). DLX5 and DLX6 (cfa14: 25014704-25033706) were selected for sequencing due to their roles as transcription factors in craniofacial development and the similar phenotypes observed in mutant mice [20]–[22]. The coding regions and intronic regions with high conservation across species of DLX5 and DLX6 were sequenced in one CP1 case and one unaffected control NSDTR. One intronic nucleotide insertion (25032667-25032667insG) was identified within DLX5 in both the CP1 case and unaffected control NSDTRs when compared to the CanFam 2.0 Boxer reference sequence [19]. Upon further investigation in additional NSDTRs, it was observed that this insertion does not segregate with the phenotype and is likely breed specific. A 2056 bp insertion was identified within a highly conserved region of DLX6 intron 2 at cfa14.25016716 in the CP1 case when compared to the unaffected control and reference sequence. The insertion is 82 bp into intron 2 and is flanked by a 13 bp target site duplication. A BLAST search identified 99% similarity with a LINE-1 element (GenBank AC187025.7) [23].

### Transcription of the LINE-1 Element

**Figure 3:**
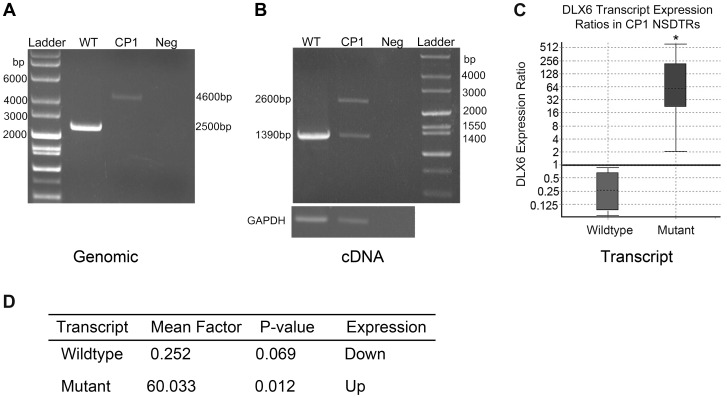
PCR amplification and expression analysis of LINE insertion in CP1 and WT NSDTRs. A. Gel image of long range PCR amplification of the DLX6 intronic LINE-1 insertion from genomic DNA of an unaffected NSDTR (WT; 2500 bp band), CP1 NSDTR (CP1; 4600 bp band), and negative water control (Neg). CP1 has a 2.1 kb LINE insertion. B. Gel image of RT-PCR amplification of DLX6 from WT and CP1 cDNA. CP1 expresses both a wildtype (1390 bp) and mutant (2600 bp) transcript. The mutant transcript has a 1.2 kb insertion. GAPDH was used to control for cDNA concentrations. C. Relative DLX6 gene expression ratios by transcript of cerebral cortex cDNA from 3 neonatal CP1 NSDTRs compared to 3 neonatal WT NSDTRs. Boxes represent the interquartile range, and the dotted lines within represent median gene expression. Whiskers of the boxplot represent minimum and maximum observations. Relative expression levels were normalized to the housekeeping gene, B2M. Statistical significance is reported as p<0.05(*). D. Summary of relative expression of wildtype and mutant DLX6 transcripts of CP1 NSDTRs compared to WT NSDTRs. Fold change and p-values were calculated using REST2009 [24].

Transcript analysis was performed in cDNA from cerebral cortex for both DLX5 and DLX6. DLX5 and DLX6 transcripts were Sanger sequenced in one neonatal CP1 NSDTR with the LINE-1 insertion and compared to one neonatal unaffected control NSDTR. No polymorphisms were identified within DLX5. cDNA from the CP1 NSDTR showed both the wildtype DLX6 transcript and a larger transcript, which contained 1281 base pairs of the intronic LINE-1 insertion (Figure 3B).

In order to quantify the amount of both wildtype DLX6 transcript and the larger mutant DLX6 transcript, real-time PCR of DLX6 from cerebral cortex cDNA was performed in 3 neonatal CP1 NSDTRs and 3 neonatal unaffected control NSDTRs. REST analysis indicated that in CP1 NSDTRs, when compared to control NSDTRs, the DLX6 wildtype transcript was downregulated by a mean factor of 0.252 (p = 0.069), while the DLX6 mutant transcript was significantly upregulated by a mean factor of 60.033 (p = 0.012; Figures 3C and 3D) [24]. Real-time PCR was also performed on DLX5 in the same samples. There was no significant change in DLX5 expression levels between CP1 cases and unaffected controls in the tissues examined.

**Figure 4:**
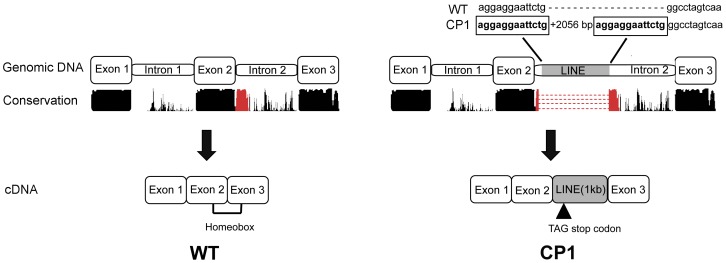
Schematic illustration of genomic and cDNA DLX6 gene structure in unaffected (WT) and CP1 NSDTRs (CP1). Nucleotides boxed and in bold are the 13 base pair target site duplication identified as part of the DLX6 LINE-1 insertion. Conservation represents the UCSC genome browser comparative genomics conservation track of human, dog, mouse, and rat sequence conservation. The region of conservation represented in red is the region disrupted by the LINE-1 insertion. Image is not drawn to scale.

Sequence analysis and translation of the approximately 1.2 kilobase LINE-1 insertion predicts an in frame stop codon after the insertion of a new exon (Figure 4). A premature stop codon is predicted to truncate 17 amino acids from the 60 amino acid functional DNA binding homeodomain.

### Segregation Analysis and Allele Frequency

**Figure 5:**
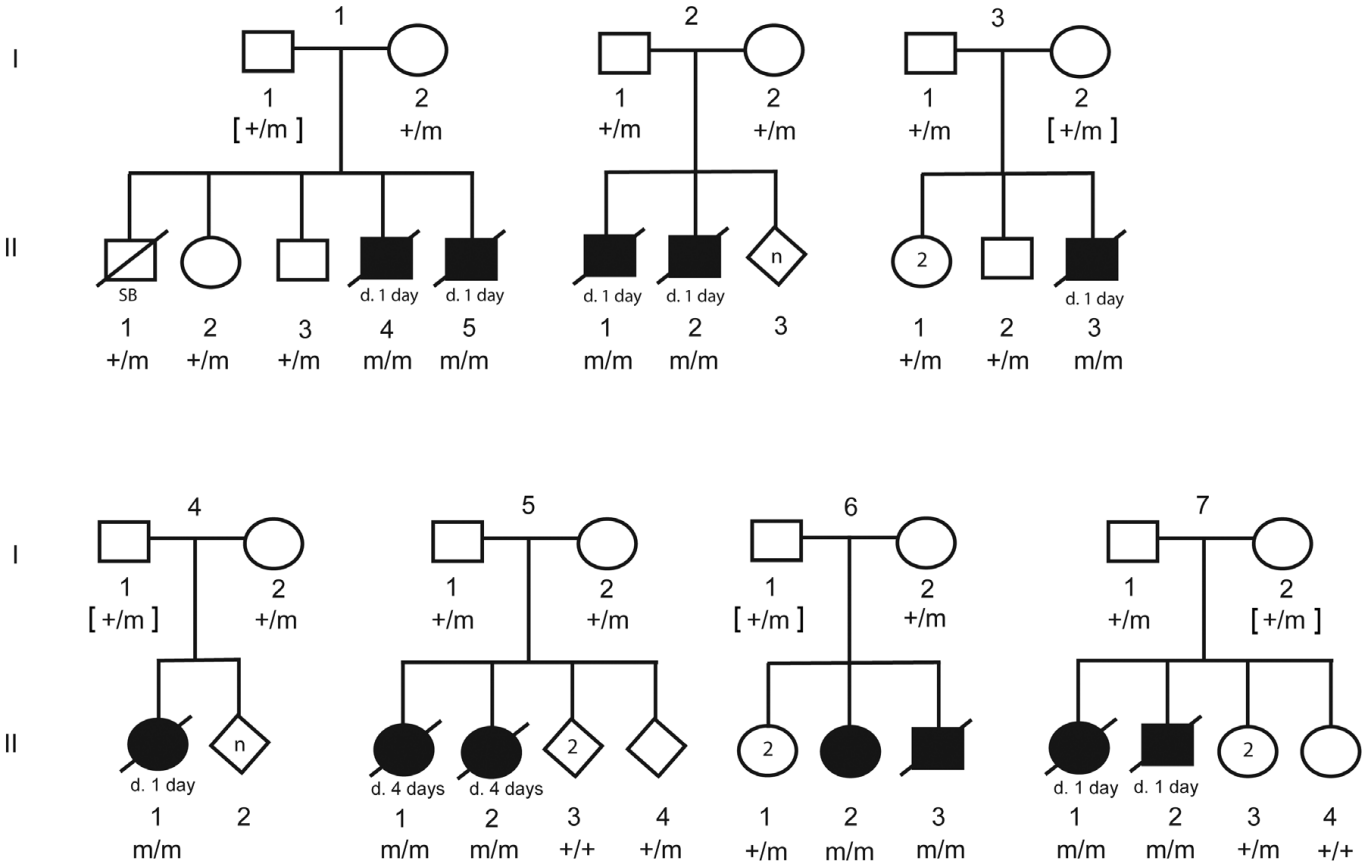
Pedigree of 7 CP1 NSDTR families depicting segregation of the mutant allele with the LINE-1 element insertion. Filled symbols represent NSDTRs with the CP1 phenotype. Diagonal lines indicate that the NSDTR is deceased. “+” represents wildtype allele. “m” represents the mutant allele. [ ] genotypes were inferred if DNA was not available.

In order to investigate segregation of the DLX6 LINE-1 insertion, PCR genotyping was performed on available DNA from parents, littermates, and the 12 CP1 NSDTRs (Figure 5). All 12 CP1 NSDTRs with the associated haplotype were homozygous for the LINE-1 insertion or mutant allele. Within families of the 12 CP1 NSDTRs, nine parents and 17 littermates were available for genotyping. Nine parents and 14 littermates were heterozygous for the mutant allele, while the 3 remaining littermates were homozygous for the wildtype allele. This indicates that the DLX6 LINE-1 insertion segregates both with the phenotype and with an autosomal recessive mode of inheritance.

Additional dogs were PCR genotyped to determine the allele frequency of the DLX6 LINE-1 insertion (Table 1). Within the NSDTR breed, 96 dogs were genotyped and 80 NSDTRs did not carry the insertion, while the remaining 16 NSDTRs were heterozygous for the insertion. To determine if the insertion was shared among other breeds, 35 affected dogs from 20 other breeds and 284 unaffected dogs from 69 breeds were genotyped. No carriers were identified. This is consistent with a fully penetrant autosomal recessive causative mutation that is private to the NSDTR breed.

**Table 1:** Summary of allele frequencies of genotyping results.

Sample	N	Allele Frequency^a^
NSDTRs	96	17%
Non NSDTRs with cleft	35	0%
Non NSDTRs	284	0%

a Allele frequencies indicate the number of individuals with at least one copy of the mutant allele.

### Screening of Human Cleft Palate Cases

To determine if variation in DLX5 or DLX6 contributed to cleft palate in humans, a cohort of patients with a variety of manifestations of cleft palate were sequenced. Sequencing of 59 patients with nonsyndromic CP (NSCP) and 92 patients with nonsyndromic clefting of the lip and palate (NSCLP) identified 7 novel intronic or 3′UTR variants (Table 2). The intronic variant at chr9: 96635849 was present in 1.25% of chromosomes and was absent from 1000 Genomes (p = 0.03) [25]. However, none of the variants was significantly associated when correcting for multiple comparisons. To test for overtransmission of a common allele to affected offspring, a set of 362 case-parent trios from the US were genotyped across a subset of SNPs (rs2272280, rs3801290, and rs3213654) and tested with TDT analysis. These SNPs were not significantly associated with NSCLP or NSCP (p>0.4, data not shown).

**Table 2:** Minor allele frequency of DLX5 and DLX6 variants among human cleft cohort and control cohort.

					NS CLP	NS CP	NS CP (US Only)	Syndromic CP	Pierre Robin Only	1000 Genomes (EUR)	NHLBI ESP (EUR)
					N = 92	N = 59	N = 46	N = 46	N = 30	N = 379	N = 4300
Gene	Position (Hg19)	rs ID	Location	Alleles	MAF (%), p-value	MAF (%)	MAF (%), p-value	MAF (%)	MAF (%)	MAF (%)	MAF (%)
DLX6	96635741	rs185935123	intronic	C/T	1.3%	0.0%	0.0%	1.8%	3.8%	0.4%	–
	96635849		intronic	G/A	**1.25%, p = 0.03**	0.0%	0.0%	0.0%	0.0%	–	–
	96636865	rs1207727	intronic	A/C	3.2%	8.3%	11.0%	6.4%	6.3%	5.8%	–
	96637047	rs141424124	Q178Q	G/A	0.6%	0.0%	0.0%	0.0%	0.0%	0.0%	2.4%
	96637185		intronic	C/T	0.0%	0.9%	0.0%	0.0%	0.0%	–	–
	96637654		intronic	A/G	0.6%	0.0%	0.0%	0.0%	0.0%	–	–
	96638021	rs1207728	intronic	G/C	20.1%	25.0%	25.6%	20.7%	21.7%	19.7%	–
	96638045	rs1004278	intronic	G/C	37.4%	26.8%	27.9%	26.3%	26.1%	30.5%	–
	96638237	rs3801290	intronic	A/G	36.8%	26.8%	27.9%	26.3%	26.1%	30.5%	–
	96638267	rs3801289	intronic	A/C	29.1%	37.5%	36.0%	41.3%	34.8%	36.7%	–
	96638763		intronic	T/C	0.0%	0.0%	0.0%	1.1%	1.9%	–	–
	96639323		P282P	A/G	0.6%	1.0%	1.3%	1.2%	1.9%	–	0.2%
	96639368	rs3213654^a^	3′ UTR	A/G	**1.15%, p = 0.04**	2.9%	1.3%	2.4%	1.9%	0.0%	0.05%
	96639505	rs147798666	3′ UTR	-/CTCT	20.1%	23.5%	23.7%	19.0%	21.2%	–	–
	96639654		3′ UTR	C/A	0.6%	0.0%	0.0%	0.0%	0.0%	–	–
	96639683		3′ UTR	C/T	0.0%	1.0%	1.4%	0.0%	0.0%	–	–
	96639753	rs34606656	3′ UTR	C/T	37.9%	27.1%	29.2%	25.6%	26.0%	30.5%	–
	96640130	rs2272280^b^	3′ UTR	C/T	1.4%	2.7%	**1.16%, p = 0.01**	3.3%	1.9%	0.0%	–
	96640311	rs191071332	3′ UTR	T/C	2.7%	0.9%	1.2%	1.1%	0.0%	1.2%	–
DLX5	96651698		intronic	C/T	0.6%	0.0%	0.0%	0.0%	0.0%	–	–
	96651406	rs1207733	intronic	G/A	7.2%	7.0%	9.1%	1.1%	1.9%	6.1%	–
	96651391	rs193302639	intronic	G/A	0.6%	0.0%	0.0%	0.0%	0.0%	0.0%	–
	96650408		intronic	C/T	0.0%	0.0%	0.0%	1.1%	0.0%	–	–
	96650342		I192M	C/G	0.0%	0.0%	0.0%	1.1%	1.9%	–	0.0%
	96650216	rs35273378	S234R	C/A	1.2%	1.8%	2.4%	1.1%	1.9%	0.0%	1.2%
	96650098	rs199678276	P274A	C/G	0.0%	0.0%	0.0%	0.0%	0.0%	0.0%	0.0%
	96649884	rs112772747	3′ UTR	-/C	29.3%	30.2%	30.3%	29.5%	32.7%	–	–

N Number of individuals sequenced; CP Cleft palate; NS Nonsyndromic; CLP Cleft lip and palate.

– Genotypes counts not available.

a,b These SNPs have minor allele frequencies from 7–11% in non-European populations.

SNPs with MAF listed in bold are statistically significant when compared to MAF of 1000 genomes and ESP databases [25], [26].

The screening of 31 patients with syndromic CP identified 4 novel rare variants, two of which were protein coding (Table 2). These include a synonymous variant at p.Phe282 and a novel missense variant in a patient with PRS (DLX5: p.Ile192Met; NM_005221.5:c.576C>G). The missense variant was predicted to be damaging by SIFT, but was inherited from the affected individual's unaffected mother. However, this variant affects a highly conserved residue of the DNA-binding domain of DLX5 that is conserved across vertebrates and was absent from both the 1000 Genomes and NHLBI ESP databases [25], [26]. An additional 15 patients with Pierre Robin sequence were sequenced, but no additional mutations were identified.

## Discussion

A naturally occurring animal model for PRS was discovered in twelve cases of CP1 NSDTRs, which exhibit relative mandibular brachygnathia and cleft palate. A genome-wide association study within NSDTRs with CP identified a shared 5.1 Mb homozygous haplotype among 12 CP1 cases with relative mandibular brachygnathia. From the associated interval, DLX5 and DLX6 were selected as regional candidate genes based on their roles in development. Sequencing of these regional candidate genes in NSDTRs identified an intronic LINE-1 insertion within DLX6 that segregates with the phenotype in the breed. This discovery prompted the sequencing of the same genes in a human cohort of CP cases, which identified a damaging missense mutation within the homeobox of DLX5 in a patient with PRS was identified.

The regional candidate genes, DLX5 and DLX6, make up a pair of convergently transcribed homeobox containing transcription factors [27]. They are involved in patterning of craniofacial structures, inner ear, limb, and brain development with roles in chondrocyte and osteoblast differentiation [21], [28], [29]. The functions of Dlx5 and Dlx6 were studied by targeted inactivation of murine homologs. Developmental expression of both genes was observed in the first pharyngeal arch, brain, and skeleton [27]. Single gene mutants (Dlx5^−/−^ and Dlx6^−/−^) were perinatal lethal and resulted in brain, bone, and inner ear defects, with craniofacial abnormalities including a cleft palate, hypoplastic condylar process, and shortened mandible [20]–[22]. Double mutants (Dlx5^−/−^;DLX6^−/−^) exhibited more severe craniofacial, inner ear, and bone defects, and were a phenocopy of split hand/foot malformation [28]. They also exhibited homeotic transformation of the mandible into a maxilla indicating that these genes were responsible for normal patterning of the mandible [21], [28], [29].

Phenotypic similarity between CP1 NSDTRs, the PRS patient, and mutant mice are observed with the changes in the condylar process, cleft palate, and relatively shortened mandibles. There may be associated condylar hypoplasia in PRS, but this was not investigated in the PRS patient [30], [31]. Shortened mandibles have been observed to cause cleft palate in mouse studies [7], [32]–[34]. Palatal development is a highly regulated sequence of events involving the repositioning of the palatal shelves from a lateral to horizontal orientation over the tongue. During this process, the tongue repositions by elongation of the mandible, allowing for reorientation of the palatal shelves (reviewed in [5]). If the mandible does not elongate, the tongue cannot reposition, which in turn obstructs palatal shelf elevation and leads to a cleft palate [7]. This supports the role of DLX5 and DLX6 in the phenotype observed in the PRS patient and CP1 NSDTRs. This also suggests that the CP1 NSDTRs are naturally occurring animal models for PRS because, although other disorders may be associated with cleft palate and micrognathia, these dogs lack additional abnormalities [35].

DLX5 and DLX6 contain homeoboxes that regulate transcription by binding to specific DNA sequences. Homeoboxes are highly conserved nucleotide sequences of 180 base pairs. Point mutations located within the homeobox give rise to disease at a higher frequency than mutations located within the rest of the gene and often show a dominant effect due to haploinsufficiency [36]. DLX5 is sensitive to haploinsufficiency because, although Dlx5^+/−^ mice appear normal, closer investigation indicates that they have a decreased bone mineral density [20], [37]. Mutations within the homeobox may also affect proper structural folding of the protein and DNA binding specificity leading to a mutant phenotype [38]. This is observed in a homozygous missense mutation in the homeobox of DLX5 in two affected family members in a consanguineous pedigree with split-hand/foot malformation (SHFM) [39]. The affected individuals were noted to have clefts of the hands, but no cleft palate or craniofacial abnormalities with the exception of a mildly pronounced forehead. The heterozygous missense mutation identified in the PRS patient (NM_005221.5:c.576C>G) is located within the homeobox sequence of DLX5, which supports it contribution to the observed phenotype.

The DLX5 heterozygous missense mutation within the PRS patient does not segregate with a Mendelian mode of inheritance, but this is not expected since PRS is a complex trait. PRS was not apparent in the mother, but she may have exhibited subtle craniofacial abnormalities that went unnoticed. This likely complex inheritance suggests a role for additional, not yet identified loci or environmental factors. Support for this is observed when mice with heterozygous expression of a Dlx5 null allele and targeted inactivation of a transcription factor responsible for clefting in people, Msx1, exhibit cleft palate (Dlx5^−/+^; Msx1^−/−^) [40]. However, when expression of both genes is disrupted (Dlx5^−/−^; Msx1^−/−^), mice have no cleft palate.

Nine noncoding sequence variants were identified within DLX5 and DLX6 of human patients with orofacial clefts. Functional analysis of the nine variants may provide further insight into the possible contribution of these genes to a cleft palate phenotype. Noncoding regions have important regulatory functions as they have been observed to disrupt gene expression. Reduced DLX5 and DLX6 expression was observed in an autistic patient with a SNP in the DLX5 and DLX6 intergenic region [41]. Hearing loss and craniofacial defects including cleft palate and micrognathia have been observed in the deletion of a DLX5 and DLX6 enhancer region [42]. The LINE-1 insertion within DLX6 of the CP1 NSDTRs is also located within a noncoding region that is highly conserved. According to the UCSC genome browser, the HMR conserved transcription factor binding site regulation track indicates that the LINE-1 insertion identified within DLX6 CP1 NSDTRs disrupts a binding domain for SUZ12. SUZ12 is a long noncoding RNA that encodes a core of the polycomb repressive complex2 that has regulatory functions in the developing embryo [43]. The exact interaction of SUZ12 and DLX6 is not yet known, indicating that the LINE insertion may do more than disrupt transcription.

LINE elements are transposable elements that comprise 21% of the human genome, 16% of the dog genome, and often insert into intronic regions [44], [45]. Intronic LINE element insertions are observed to cause disease in Duchenne-like muscular dystrophy and chronic granulomatous disease through the introduction of a new exon [46]–[48]. cDNA sequence from CP1 NSDTRs homozygous for the LINE-1 insertion indicate that 1 kb of the LINE element is spliced into the DLX6 transcript. Premature protein truncation of DLX6 is predicted due to an in frame stop codon after the formation of a new exon. Both the Dlx6 mutant mice (Dlx6^−/−^) and CP1 NSDTRs phenotypes are the result of truncating the same 3′ sequence of the homeodomain [22].

The LINE insertion disrupts transcription of DLX6 within CP1 NSDTRs and leads to downregulation of wildtype DLX6 transcript when compared to unaffected NSDTRs. As a result,, only 25% of the normal expression levels are produced. The reduced expression of wildtype DLX6 transcript is not enough to prevent CP and the mandibular abnormalities. DLX5 and DLX6 expression levels have been observed to vary based on the timepoint and tissue examined with complex transcriptional regulation from tissue specific enhancers and noncoding RNAs [20], [21], [27], [28], [42], [49]–[51]. It is possible that the LINE insertion works to disrupt appropriate timing of tissue specific expression since it is inserted into a highly conserved region within intron 2. Although not statistically significant, expression analysis from additional biological replicates from the correct tissue during the correct embryonic timepoint would likely yield significant values in the CP1 NSDTRs.

This discovery provides a genetic tool for the NSDTR breeder who wishes to avoid producing cleft palate affected puppies. The LINE insertion identified within the CP1 NSDTRs is unique to a subset of cases with CP within the breed and cannot be used as a tool to prevent against all genetic causes of CP. CP disease heterogeneity is observed in the NSDTR breed as 2 of the 14 CP cases did not possess relatively shortened mandibles and the associated LINE-1 insertion. This is unexpected in a purebred dog breed with few founders where the inbreeding coefficient is 0.26 [52]. This indicates that even in relatively genetically homogenous samples, heterogeneity and a complex etiology that mimics human cleft cases such as PRS is observed. This highlights the promise of the dog as an animal model for birth defects to identify multiple genes and/or pathways involved in craniofacial development.

In summary, identifying a mutation in an animal model with naturally occurring birth defects has enabled the identification of new candidate genes for PRS in people. This supports the continued use of the naturally occurring birth defects found within dogs and their unique genetic background to further our understanding of commonly occurring birth defects in people.

## Materials and Methods

### Ethics Statement

The use of samples involving human participants was approved by the institutional review board at the University of Iowa (approval #s 199804080 and 199804081). Informed consent was obtained and all clinical investigation was conducted according to the principles expressed in the Declaration of Helsinki. The collection of canine samples used in this study was approved by the University of California, Davis Animal Care and Use Committee (protocol #16892).

### Canine Samples and DNA Extraction

Blood and tissue samples from NSDTRs with cleft palate (n = 14), healthy littermates (n = 24), parents (n = 11), unaffected NSDTRs (n = 153), and dogs with cleft palate across 20 breeds (n = 35) were collected from privately owned animals. When available, tissue was collected at post mortem examination and flash frozen. The evaluations of orofacial clefts were performed by visual inspection of affected dogs. Blood samples from unaffected dogs (n = 284) across 69 other breeds were collected from the William R. Pritchard Veterinary Medical Teaching Hospital. Genomic DNA was extracted from EDTA whole blood and tissue samples using Gentra Puregene DNA purification extraction kit (Qiagen, Valencia, CA).

### Genome-Wide Association Study

Genome-wide SNP genotyping was performed with 14 cases and 72 controls using the Illumina CanineHD BeadChip (Illumina, San Diego, CA) with 173,662 markers. Samples had a genotyping call rate of ≥90%. 63,195 SNPs were excluded due to a minor allele frequency of ≤0.05 and 2,994 SNPs were excluded for a missing call rate of ≤10%. Chi-square analysis was performed using Plink [53]. 100,000 permutations were performed to correct for multiple tests. To determine if population stratification was present, quantile- quantile plots were generated in R using Plink output data [54]. The genomic inflation factor was also calculated using Plink.

### CT Imaging

High-resolution micro-computed tomography (micro-CT) was used to evaluate craniofacial structures in 4 CP1 NSDTRs that were homozygous for the LINE-1 insertion, and in 3 normal NSDTRs homozygous for the wildtype allele. Samples were imaged at the Center for Molecular and Genomic Imaging (UC Davis). The skulls were kept in zip-lock bags and allowed to warm to the CT scanner temperature (29°C) inside of a custom plastic holder. CT images were obtained on the Centers MicroXCT-200 specimen CT scanner (Carl Zeiss X-ray Microscopy). The CT scanner has a variable x-ray source capable of a voltage range of 20–90 kV with 1–8W of power. Samples were mounted on the scanners sample stage, which has submicron level of position adjustments. Scan parameters were adjusted based on the manufacturer's recommended guidelines: source and detector distances were 108 mm and 35 mm, respectively; the manufacturers LE4 custom filter was used for beam filtration; the voltage and power were set to 70 kV and 8W, respectively; and 1600 projections were acquired over 360-degrees with an exposure time of 1.5 s. Images were reconstructed on an isotropic voxel grid with 51.1507 microns per edge. Digital TIFF images were imported into Amira 5.4.5 (Visualization Sciences Group, FEI) For all specimens, tridimensional reconstructive (3D) images were generated to assess the spatial relationship of the bones. 3D length measurements were performed using the 3D length tool. Visual inspection of the micro-CT images was performed to identify any abnormalities.

### PCR Amplification of Microsatellites and Linkage Analysis

Four regional microsatellites were mined from the UCSC genome browser (CanFam 2.0) “Variation and Repeats” database within cfa14: 24.2 Mb–29.3 Mb. Fluorescently labeled microsatellite primers were designed (Table S4) using Primer3 [55]. Microsatellites were PCR amplified using the following protocol: 94°C for 12 minutes, 7 cycles of 93°C for 20 seconds, 65°C for 30 seconds, 72°C for 2 minutes, 5 cycles of 93°C for 20 seconds, 58°C for 30 seconds, 72°C for 2 minutes, and 25 cycles of 93°C for 20 seconds, 55°C for 30 seconds, 72°C for 2 minutes, followed by a final annealing of 72°C for 2 minutes. Genotyping was performed on an ABI 3100 Genetic Analyzer (Applied Biosystems, CA) in 8 CP1 NSDTRs and 32 control NSDTRs. All genotypes were analyzed using STRand software [56]. LOD score analysis of microsatellite data was performed using Mendel software's LOCATION_SCORES option in 59 individuals from three pedigrees [57]. The disease trait was coded as autosomal recessive and assumed to be fully penetrant.

### Sequencing of Candidate Genes

Primers to amplify the exons, intron/exon boundaries, and regions of high conservation within the intragenic and intergenic regions of DLX5 and DLX6 were designed in Primer3 (Table S3) [55]. PCR products were amplified in one CP1 NSDTR and one unaffected NSDTR. Areas with high GC content were amplified using Invitrogen AccuPrime GC-Rich DNA Polymerase protocols (Life Technologies, Grand Island, NY). PCR products were cleaned using ExoSAP-IT and sequenced using the Big Dye terminator mix on an ABI 3100 Genetic Analyzer (Applied Biosystems, CA). Sequences were analyzed using Chromas (Technelysium, Tewantin, QLD, Australia) and Vector NTI (Informax, Frederick, MD). Sequences were aligned to each other and the Boxer reference sequence (CanFam 2.0) to identify any polymorphisms [19]. The DLX6 LINE-1 insertion was amplified using LongAmp Taq PCR Kit (New England BioLabs Ipswich, MA) and cloned using the Invitrogen TOPO TA Cloning kit (pCR2.1-TOPO vector) with One Shot TOP10 Chemically Competent E. coli. Products were isolated with the Qiaprep Spin Miniprep kit (Qiagen, Valencia, CA) and sequenced using an ABI 3100 Genetic Analyzer (Applied Biosystems, CA). LINE element sequence was identified using BLAST 2.2.28 [23].

### RNA Extraction/cDNA Sequencing

Expression of DLX5 and DLX6 was evaluated in NSDTR lymphocytes, unaffected adult beagle tissue from cerebellum, cerebral cortex, heart, kidney, liver, skeletal muscle, skin, spinal cord, spleen, testis, and thymus. Total RNA was isolated from tissue samples using Qiagen QIAamp Blood Mini Kit (Valencia, CA) tissue protocols. Adult beagle total RNA was obtained from Zyagen (San Diego, CA). RNA was synthesized into cDNA using Invitrogen Superscript III First Strand Synthesis System to RT PCR kit (Life Technologies, Grand Island, NY). GAPDH was amplified (forward primer 5′AAGATTGTCAGCAATGCCTCC 3′ and reverse primer 5′ CCAGGAAATGAGCTTGACAAA 3′) in these tissues to ensure that equivalent amounts of cDNA were added. Invitrogen 5′ prime RACE system for Rapid Amplification of cDNA ends kit (Life Technologies, Grand Island, NY) was used to sequence the 5′ prime end of DLX6 from embryo cDNA. RACE primers were designed using Primer3 (Table S3) [55]. RACE PCR products were cloned using the Invitrogen TOPO TA Cloning kit (pCR2.1-TOPO vector) with One Shot TOP10 Chemically Competent E. coli. Products were isolated with the Qiaprep Spin Miniprep kit (Qiagen, Valencia, CA) and sequenced using an ABI 3100 Genetic Analyzer (Applied Biosystems, CA).

### Genotyping

Genotyping primers were designed using Primer3 [55]. PCR genotyping was performed using a shared FAM labeled forward primer (5′ ACCATCGCTTTCAGCAAACT 3′). Unlabeled reverse primers specific for the LINE-1 insertion (5′ GCAACTAATATTCGATAAAGCAGAA 3′) and wildtype (5′ CTAGGCCCAGAATTCCTCCT 3′) were designed. The PCR program was as follows: 94°C for 12 minutes, 35 cycles of 94°C for 30 seconds, 58°C for 30 seconds, and 72°C for 45 seconds, followed by 72°C for 20 minutes. The wildtype product produced a 171 base pair product and the mutant product produced a 184 base pair product. GeneScan 500 ROX size standards were used and the reaction was analyzed on an ABI 3100 Genetic Analyzer (Applied Biosystems, CA). 96 unrelated unaffected NSDTRs and 284 unaffected dogs across 69 breeds were genotyped for the insertion. Nonsyndromic cleft palate cases (n = 35) across 20 breeds were genotyped. All genotypes were analyzed using STRand software [56].

### Real Time Quantitative PCR

Primer sequences were generated using Primer3Plus (http://primer3plus.com/). A shared forward primer (5′aaactcagtacctggcccttc 3′) was designed with reverse primers unique to wildtype (5′ccatatcttcacctgtgtttgtg 3′) and mutant (5′aaactcagtacctggcccttc 3′). Semi quantitative PCR using AmpliTaq Gold DNA Polymerase was performed to test the quality of cDNA and primers, to confirm product size and to check for the presence of genomic DNA contamination. Real-time PCR was performed using the Rotor-Gene SYBR Green PCR Kit (QIAGEN, Valencia, CA) using a 2-step cycle protocol (45 cycles; Initial denaturation-5 minutes at 95°C; Annealing- 15 seconds at 95°C; Extension- 90 seconds at 95°C; Final Melt curve) on the Rotor Gene Q real-time PCR system. Cerebral cortex from 3 neonatal unaffected NSDTRs and 3 neonatal CP1 NSDTRs were run in triplicates with each replicate containing 0.2 ng template cDNA. cDNA was prepared as described above. All data was normalized to the housekeeping gene B2M5 [58]. Amplification and takeoff values were analyzed and graphed by REST2009 to determine any significant expression differences in DLX5 and DLX6 transcript levels between control and affected cDNA samples [24].

### Human Samples

The case cohort consisted of 92 samples from individuals with nonsyndromic cleft lip with cleft palate (NSCLP) from the US, 59 samples from individuals with nonsyndromic cleft palate (NSCP) from the US and the Philippines, and 46 samples from individuals with cleft palate syndromes. The 46 syndromic samples consisted of 30 samples from individuals with PRS and 16 samples from individuals with additional congenital anomalies including club foot, hearing loss, heart disease, and intellectual disability. 60 unrelated CEPH samples (CEU) were used as controls.

### Sequencing of Human Samples

Primers were designed with Primer3 to cover the complete gene region of DLX6 and all exons of DLX5 [55]. Primer sequences and annealing temperatures are available in Table S5. The first exon of DLX6 was sequenced using internal primers. PCR products were sequenced on an ABI 3730XL (Functional Biosciences, Inc., Madison, WI). Chromatograms were transferred to a Unix workstation, base-called with PHRED (v.0.961028), assembled with PHRAP (v. 0.960731), scanned by POLYPHRED (v. 0.970312), and viewed with the CONSED program (v. 4.0). The functional effects of variants were predicted using the Variant Effect Predictor [59]. Genotypes from European or Asian populations available from the 1000 Genomes Project and the NHLBI Exome Sequencing Project were used as controls [25], [26]. Fisher's exact test, implemented in STATA (v12.1), was used to test for differences in allele frequencies between NSCLP and NSCP cases and controls [60]. For insertions or deletions where allele frequencies were unavailable, we sequenced a set of 60 unrelated CEPH samples.

### Genotyping of Human Samples

Three SNPs (rs2272280, rs3801290, and rs3213654) were genotyped in 362 case-parent trios (1090 individuals) with nonsyndromic cleft lip with or without cleft palate (NSCL/P) from the US using TaqMan SNP Genotyping Assays (Life Technologies, Grand Island, NY) on the ABI Prism 7900HT, and were analyzed with SDS 2.3 software (Applied Biosystems, Foster City, CA). The Family Based Association Test (FBAT) in STATA (v12.1) was used to test for association these NSCL/P case-parent trios [60].

## Supporting Information

Table S1 Location of microsatellite markers on CFA14, calculated LOD scores, and recombination fraction (**θ**). Genomic location based on Can Fam 2.0 assembly.
(DOCX)

Table S2 Average lengths of neonatal NSDTRs mandibles. Length measurements of the mandible are taken from the angular process to rostral tip of the mandibular body.
(DOCX)

Table S3 Genes located within associated interval on chromosome 14. Genomic locations are based on the Can Fam 2.0 assembly and refer to chromosome 14 base pair locations. Genes listed were identified using the UCSC genome browser RefSeq gene annotation track.
(DOCX)

Table S4 Primers and annealing temperatures for sequencing of canine samples. Genomic locations are based on the Can Fam 2.0 assembly and refer to chromosome 14 base pair locations. ^a^ LINE insert begins at cfa14.25016704. S – primers used for sequencing.
(DOCX)

Table S5 Primers and annealing temperatures for sequencing of canine samples. Genomic locations are based on the hg19 assembly and refer to chromosome 7 base pair locations. S – primers used for sequencing.
(DOCX)
