# Supplementary material for: A LINE-1 Insertion in DLX6 Is Responsible for Cleft Palate and Mandibular Abnormalities in a Canine Model of Pierre Robin Sequence
Source: PLoS Genet. 2014 Apr 3;10(4):e1004257. doi: 10.1371/journal.pgen.1004257 (PMC3974639; doi:10.1371/journal.pgen.1004257)
Supplement: Table S1 — Location of microsatellite markers on CFA14, calculated LOD scores, and recombination fraction (θ). Genomic location based on Can Fam 2.0 assembly. (DOCX) [file pgen.1004257.s001.docx]

Supplemental Table 1. Location of microsatellite markers on CFA14, calculated LOD scores, and recombination fraction (**θ).**

| CFA14 Marker | Genomic Location^a^  CFA14* | LOD Score | **Θ** |
| --- | --- | --- | --- |
| 1423B | 23436691 | 1.48 | 0.05 |
| 1423D | 23685492 | 2.48 | 0 |
| 1424I | 24586440 | 0.00 | 0.2 – 0.5 |
| 1425A | 25006375 | 3.18 | 0 |

^a^ Genomic location based on Can Fam 2.0 assembly.
