# Supplementary material for: A LINE-1 Insertion in DLX6 Is Responsible for Cleft Palate and Mandibular Abnormalities in a Canine Model of Pierre Robin Sequence
Source: PLoS Genet. 2014 Apr 3;10(4):e1004257. doi: 10.1371/journal.pgen.1004257 (PMC3974639; doi:10.1371/journal.pgen.1004257)
Supplement: Table S2 — Average lengths of neonatal NSDTRs mandibles. Length measurements of the mandible are taken from the angular process to rostral tip of the mandibular body. (DOCX) [file pgen.1004257.s002.docx]

Supplemental Table 2. Average lengths of neonatal NSDTRs mandibles

| Samples | Average Length (mm)  CFA14* |
| --- | --- |
| CP1 NSDTRs | 27.03 |
| WT NSDTRs | 32.49 |

**DIfference 5.46 mm**

Length measurements of the mandible are taken from the angular process to rostral tip of the mandibular body
