# Supplementary material for: A LINE-1 Insertion in DLX6 Is Responsible for Cleft Palate and Mandibular Abnormalities in a Canine Model of Pierre Robin Sequence
Source: PLoS Genet. 2014 Apr 3;10(4):e1004257. doi: 10.1371/journal.pgen.1004257 (PMC3974639; doi:10.1371/journal.pgen.1004257)
Supplement: Table S3 — Genes located within associated interval on chromosome 14. Genomic locations are based on the Can Fam 2.0 assembly and refer to chromosome 14 base pair locations. Genes listed were identified using the UCSC genome browser RefSeq gene annotation track. (DOCX) [file pgen.1004257.s003.docx]

Supplemental Table 3. Genes located within associated interval on chromosome 14

| Gene Symbol | Gene Description | Genomic Location |
| --- | --- | --- |
| DYNC1/1 | dynein, cytoplasmic 1, intermediate chain 1 | 23914934-24226072 |
| SLC25A13 | solute carrier family 25 (aspartate/glutamate carrier), member 13 | 24243683-24428843 |
| SHFM1 | split hand/foot malformation (ectrodactyly) type 1 | 24714174-24737126 |
| ACN9 | ACN9 homolog | 25101642-25169474 |
| DLX6 | distal-less homeobox 6 | 25014701-25019783 |
| DLX5 | distal-less homeobox 5 | 25029340-25033703 |
| UQCR11 | ubiquinol-cytochrome c reductase, complex III subunit XI | 25235673-25236334 |
| LRRC37A | leucine rich repeat containing 37 | 25266819-25275154 |
| TAC1 | tachykinin, precursor 1 | 25580697-25588967 |
| ASNS | asparagine synthetase (glutamine-hydrolyzing) | 25666695-25679126 |
| C1GALT1 | core 1 synthase, glycoprotein-N-acetylgalactosamine 3-beta-galactosyltransferase | 25812586-25850889 |
| COL28A1 | collagen, type XXVIII, alpha 1 | 25941958-26101940 |
| MIOS | missing oocyte, meiosis regulator | 26119803-26155175 |
| RPA3 | replication protein A3 | 26179745-26183584 |
| GLCCI1 | glucocorticoid induced transcript 1 | 26505072-26605846 |
| ICA1 | islet cell autoantigen 1, 69kDa | 26621950-26765628 |
| NXPH1 | neurexophilin 1 | 26904996-27199125 |
| RPL17 | ribosomal protein L17 | 28774693-28775290 |
| NOVFA4 | NADH dehydrogenase (ubiquinone) 1 alpha subcomplex | 28835609-28842234 |
| CENPV | centromere protein V | 29207511-29208166 |
| THSD7 | thrombospondin, type I, domain containing 7A | 29292957-29717573 |

Genomic locations are based on the Can Fam 2.0 assembly and refer to chromosome 14 base pair locations. Genes listed were identified using the UCSC genome browser RefSeq gene annotation track.
