# Supplementary material for: A LINE-1 Insertion in DLX6 Is Responsible for Cleft Palate and Mandibular Abnormalities in a Canine Model of Pierre Robin Sequence
Source: PLoS Genet. 2014 Apr 3;10(4):e1004257. doi: 10.1371/journal.pgen.1004257 (PMC3974639; doi:10.1371/journal.pgen.1004257)
Supplement: Table S4 — Primers and annealing temperatures for sequencing of canine samples. Genomic locations are based on the Can Fam 2.0 assembly and refer to chromosome 14 base pair locations. a LINE insert begins at cfa14.25016704. S – primers used for sequencing. (DOCX) [file pgen.1004257.s004.docx]

Supplemental Table 4. Primers and Annealing Temperatures for Sequencing of Canine Samples

| **Gene** |  |  | **Genomic location** | **Sequence** | **Product Size** | **Annealing Temperature** |
| --- | --- | --- | --- | --- | --- | --- |
| **DLX5** | exon 1 | F | 25033830 | GCAATGCTTTGTTGTGCTAAA | 851 | 57 |
|  |  | R | 25033000 | CTGGACCCTGCAACAGAGAG |  |  |
|  |  | S1 [R] | 25033263 | CAGTACCAGTACCACGGCGT |  |  |
|  |  | S2 [F] | 25033576 | CCACAGCCATGTCTGCTTAG |  |  |
|  | intron1 | F | 25031454 | AAAGGCCAGGTAGTTTGGTT | 689 | 59 |
|  |  | R | 25032122 | CGACTTTGAGAAATTCGTCCT |  |  |
|  | exon 2 | F | 25031359 | GCGTGGGTCAAAAAGAAAAC | 600 | 58 |
|  |  | R | 25030785 | CTCCAGGCTTCCAGAGTGTC |  |  |
|  | intron 2 | F | 25030980 | CGCGAGAAACTCACAGACAG | 755 | 58 |
|  |  | R | 25030245 | GGACTCAGGAGGCTTCAGTTT |  |  |
|  | exon 3 part 1 | F | 25030086 | ACCCGAGATGCCTCCAGT | 578 | 59 |
|  |  | R | 25029531 | GGGGTCCTTTGAAATGCAATA |  |  |
|  | exon 3 part 2 | F | 25029818 | CTCCGACCTCCAACCAGTC | 659 | 59 |
|  |  | R | 25029181 | CACGTGTACAGTTTTGCATCC |  |  |
|  | cDNA | F | 25033462 | AGAAGGGTCCCCAGCATC | 1474 | 60.5 |
|  |  | R | 25029718 | CAGCGGGTGCTGTAAGGAG |  |  |
|  |  | S1 [F] | 25031227 | GGCAAACCAAAGAAAGTTCG |  |  |
| **DLX6** | 5' | F | 25013350 | CCAGAAAGTAAAAATAACTGTAAAACG | 999 | 58 |
|  |  | R | 25014327 | AAAATCTCTGCCTTAAACTGCA |  |  |
|  |  | S[R] | 25013781 | ATTGAAACCACAGAGGGAAGA |  |  |
|  | 5' RACE | GSP1 | 25016614 | CTGTGTTTGTGTCAGTCC | per RACE kit | |
|  |  | GSP2 | 25016519 | CTGCAGGCTGGAATAAATGG |  |  |
|  |  | GSP3 | 25016495 | AGGCTTCCGAATCTTTTTCC |  |  |
|  | cDNA exon 1 | F | 25014709 | CATGACTACGATGGCTGACG | 501 | 59 |
|  |  | R | 25016495 | AGGCTTCCGAATCTTTTTCC |  |  |
|  | exon 2 | F | 25016033 | AAACATCAGGGTGGCTCAAG | 807 | 60 |
|  |  | R | 25016820 | ATGCTGGATTGGATTTGCAC |  |  |
|  | intron 2 | F | 25016647 | AAGCCCAGGTATCCCTGAAA | 2359 | 59 |
|  |  | R | 25018985 | CCTTGAGCAGCCTCAGTAACT |  |  |
|  |  | S[F] | 25017152 | GGTGAGGTGGGTTTTTCTGA |  |  |
|  |  | S[R] | 25018095 | CCCAAACCTGGCATCTCTAT |  |  |
|  | LINE insertion | [S]1F | ^a^ | TCTTATGCTTTTGGGTGCAA | 2188 |  |
|  |  | [S]2F |  | TCCACGAAGGCAAAAGAAAC |  |  |
|  |  | [S]3F |  | CTGGGAAACTGTGTGGAGGT |  |  |
|  |  | [S]4F |  | GGAAACGAACTAGGGGTGGT |  |  |
|  | exon 3 | F | 25018409 | TTGGATATTGCTCCCAAAGC | 597 | 58 |
|  |  | R | 25018985 | AGTTACTGAGGCTGCTCAAGG |  |  |
|  | cDNA exon 2 + 3 | F | 25016465 | AAAACGGGGAAATCAGGTTC | 1317 | 60 |
|  |  | R | 25019641 | TGAATGCCACCAATTCACCT |  |  |
|  |  | S[F] | 25018717 | ACACGATGCAGAGACCACAG |  |  |
|  |  | S[R] | 25018699 | GTCCTGGTGTGGAGAGGAGT |  |  |
|  | 3' | F | 25019909 | TCAAGTGCCTGCAAAAGATG | 848 | 60 |
|  |  | R | 25020737 | AGCTGGAGGCTTTGTTCAGA |  |  |
|  |  | S[R] | 25020254 | AGCTAGCTTGCAAAGGGTGT |  |  |
|  |  | F | 25019430 | AACATTGCCACAAAGGGAAC | 524 | 59 |
|  |  | R | 25019934 | TTGCAGCACTAGCCTTTGTC |  |  |
| **DLX Intergenic Region** | 25022576- 25023676 | F |  | CACCAGAACGACAACCCTTT | 1120 | 59 |
|  |  | R |  | GGCATTTTGCTCTGATGACA |  |  |
|  | 25023266-25024526 | F |  | GCGATCAAATCAAACCCTGA | 1280 | 59 |
|  |  | R |  | GCCACTTGCTAGGTGGGATA |  |  |
|  |  | S1[R] | 25024196 | CCCACCCCAGGAACTAGAAT |  |  |
|  |  | S2[F] | 25023607 | CCAGGCTGCCTCAGAGTATG |  |  |
|  | 25024088-25024950 | F |  | TGGAGGGAGGCTAGGGTACT | 882 | 59 |
|  |  | R |  | TTCTTTTGTTCGCTGGGACT |  |  |
|  | 25024813-25025667 | F |  | TCCTGTGGGAAAGGTCGTAA | 874 | 59 |
|  |  | R |  | CGAGGAGAGGACCTTTAGGG |  |  |
|  | 25025308-25026277 | F |  | GCCCAGAGTTCGCTGTAAGT | 989 | 59 |
|  |  | R |  | GGCCACCTCCTTGTAGAACA |  |  |
|  | 25025933-25026806 | F |  | TCTGGGATTGTGGGATAAGG | 893 | 59 |
|  |  | R |  | CACCATGGGGATTCTCTCAG |  |  |
|  | 25026708-25027622 | F |  | AGGGCAAGGGAGAGAAACAT | 934 | 59 |
|  |  | R |  | GCAGCTTTCCTGTCTCATCC |  |  |

Genomic locations are based on the Can Fam 2.0 assembly and refer to chromosome 14 base pair locations. ^a^ LINE insert begins at cfa14.25016704 S – primers used for sequencing.
