# Supplementary material for: A LINE-1 Insertion in DLX6 Is Responsible for Cleft Palate and Mandibular Abnormalities in a Canine Model of Pierre Robin Sequence
Source: PLoS Genet. 2014 Apr 3;10(4):e1004257. doi: 10.1371/journal.pgen.1004257 (PMC3974639; doi:10.1371/journal.pgen.1004257)
Supplement: Table S5 — Primers and annealing temperatures for sequencing of canine samples. Genomic locations are based on the hg19 assembly and refer to chromosome 7 base pair locations. S – primers used for sequencing. (DOCX) [file pgen.1004257.s005.docx]

Supplemental Table 5. Primers and Annealing Temperatures for Sequencing of Human Cohort

| Gene | Region/Exon |  | Genomic Location | Sequence | Product Size | Annealing Temperature |
| --- | --- | --- | --- | --- | --- | --- |
| DLX5 | exon 1 | F | 96654239 | TGGACGAGTTAGGGTGTTACTG | 830 | 63 |
|  |  | R | 96653431 | CTCTTTGTTGGAGGGTCTGAGT |  |  |
|  | exon 2 | F | 96651777 | TATGGGCAAAGAATGAAGACTG | 501 | 57 |
|  |  | R | 96651298 | CTCCAGACCGCTGATGAATAC |  |  |
|  | exon 3 | F | 96650513 | GCATAGCTTCTTGGCGGTAG | 937 | 57 |
|  |  | R | 96649596 | TTTTGCCTTGTTGGATCTCTG |  |  |
| DLX6 | 96634927 - 96637309 | F |  | TTAAAATCTCTGCCTTAAACTGCAC | 2407 | 62 |
|  |  | R |  | TATTCAATACCAGGTCATTCATTCC |  |  |
|  |  | S1 [R] | 96636147 | CACGCAAAGTAGGCCACTGC |  | |
|  |  | S2 [F] | 96635995 | GTTAGTCCACACTGCGCTCTCC |  |  |
|  | 96636060 - 96637026 | F |  | AGGCTGTAACCCACGTTTGAC | 986 | 63 |
|  |  | R |  | GCAGGCTGGAATAAATGGTC |  |  |
|  | 96636923- 96637860 | F |  | CCGCTCCTCTGTATTATTTGCTTA | 961 | 63 |
|  |  | R |  | ATGGCTTTCAAATTAGTAGGATGG |  |  |
|  | 96637681 - 96638649 | F |  | TTTTCTGAAGATTTCCCTAAGCAC | 992 | 61 |
|  |  | R |  | TTTACACAGTCATTTTCTCCGTGT |  |  |
|  | 96638485 - 96639301 | F |  | TTATGTCTGGAGAAAGATGCTTCA | 840 | 57 |
|  |  | R |  | GTGGAGAGGAGTACCAGTGAGAAT |  |  |
|  | 96639028 - 96639899 | F |  | TATTGCTTCTAAATCCCTGAGTG | 895 | 60 |
|  |  | R |  | GAGTCCAAAATGCTTCTCTTTCAC |  |  |
|  | 96639774 - 96640718 | F |  | GTCTCTTCTCTTTGGGAGTATCCA | 967 | 63 |
|  |  | R |  | GCTGTATTATTTTGGCTGGGAAA |  |  |

Genomic locations are based on the hg19 assembly and refer to chromosome 7 base pair locations. S – primers used for sequencing.
